# An Ubuntu approach to disability and inclusive development for women with disabilities

**DOI:** 10.4102/ajod.v14i0.1600

**Published:** 2025-07-22

**Authors:** Theresa Lorenzo, Maximus M. Sefotho

**Affiliations:** 1 Department of Health and Rehabilitation Science, Division of Disability Studies, Faculty of Health Sciences, University of Cape Town, Cape Town, South Africa; 2 Department of Inclusive Practices Africa research unit, Faculty of Health Sciences, University of Cape Town, Cape Town, South Africa; 3 Health through Physical Activity, Lifestyle and Sport, Faculty of Health Sciences, University of Cape Town, Cape Town, South Africa; 4 Department of Educational Psychology, Faculty of Humanities, University of Johannesburg, Johannesburg, South Africa

**Keywords:** human scale development, disability, spirituality, Ubuntu, participatory action research, narratives, action reflection, inclusive development, social change, community-based rehabilitation

## Abstract

**Background:**

Vicious cycles of disability and poverty isolate disabled women, making it difficult for them to meet their own and their family’s human needs. Their exclusion and deprivations may be bridged through inclusive development processes. The article presents an Afro-centric approach to inclusive development that speaks to experiences of disabled women who lived in informal settlements in the Cape Town metropole, South Africa that also has relevance for marginalised and oppressed communities in the Global North.

**Objectives:**

To describe how human scale development (HSD) as a conceptual framework resonates with Ubuntu values and principles to enable individual and collective action spaces to overcome human poverties.

**Method:**

Reflexivity was done to explicate and further conceptualise an Ubuntu approach to community-based inclusive development for disabled women.

**Results:**

Analysis revealed the centrality of Ubuntu values in effecting social change through bi-directional shifts in self and collective identities, and a spirituality of disability. Themes included *Disability as a burden* (deprivation), and *Disability as a gain* (a potentiality). Five development opportunities emerged: enhanced self-identity; strengthened family life; sustained livelihood; community rehabilitation workers as brokers to facilitate access to health and social services; and information.

**Conclusion:**

Ubuntu as an African philosophy draws on indigenous knowledge systems that provide an Afro-centric approach to inclusive development of disabled women. Ubuntu promotes a reflexive, person-centred and collective approach to human development at the micro-level.

**Contribution:**

Harnessing the power of learning to listen deeply to each other’s stories facilitates the interdependence and spirituality of Ubuntu to create supportive, inclusive development.

## Introduction

**FIGURE 1:**
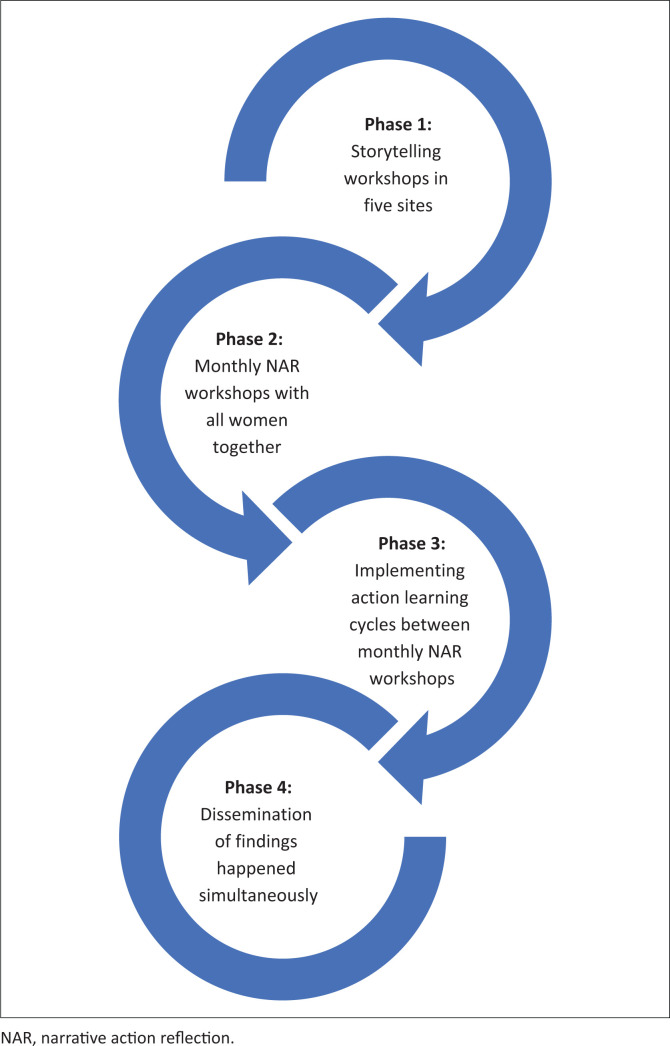
The four phases of the participatory action research process.

Disability inclusive development (DID) means that all stages of development processes are inclusive of and accessible to persons with disabilities (CBM 2015). At a national level, DID requires that all persons be afforded equal access to education, health care services, livelihoods development, family and social inclusion and other citizen rights (United Nations [UN] 2017). These ideals are difficult to attain at local community levels because persons with disability face numerous material and structural barriers in the context of poverty and underdevelopment. How disability is defined, understood and given meaning in a culture influences the extent to which essential services, support and development opportunities are made accessible to disabled persons. This article is a conceptual article on a new framework that emerged through the synthesis of the findings from empirical research that has already been published (Lorenzo 2005). The vicious cycles of disability and poverty are hard to overcome with few material resources and multiple structural barriers, especially for women with disabilities who experience heightened levels of exclusion from development opportunities such as access to adequate housing, health services, education and livelihoods development (Lorenzo & Kathard 2018). Women also face multiple forms of discrimination based on their various cultural identities, placing them at higher risk of gender-based violence, sexual abuse, neglect and exploitation and therefore in higher need of development opportunities (Lorenzo & Kathard 2018). In this article, we demonstrate the contribution of indigenous knowledge about cultural values and spiritual practices to the self and collective empowerment of disabled women. The first author carried out a participatory action research (PAR) doctoral study with disabled women who lived in informal human settlements in communities in Cape Town metropole, South Africa, over a 4-year period from 1999 to 2003 (Lorenzo 2005). Data were generated through an initial storytelling workshop in five sites and subsequent monthly narrative action reflection (NAR) workshops (Lorenzo 2010) (see Figure 1).

The purpose of the NAR workshops that were undertaken with the women was to interrupt the vicious cycles of risk and exclusion by creating action learning spaces where women with disabilities were empowered to move from being vulnerable groups to becoming resource groups that contribute to social transformation in communities (Lorenzo 2010). Using old data is justifiable when examining a past event, such as the disability framework proposed by Lorenzo (2005). We have been forthcoming about the age of the data (Ketchen, Roccapriore & Connelly 2023). We present an Ubuntu approach to disability as a framework for community-based inclusive development as identified by Lorenzo (2005). The proposal is an epistemically equitable one supporting Afrocentric Ubuntu frameworks for people with disabilities (Sefotho 2022). This article promotes ‘an Ubuntu approach to disability inclusion’ as it aligns with emerging scholarship that mainstreams Ubunutu-Botho paradigms from the global south (Mpofu & Sefotho 2024; Sefotho & Letseka 2024).

Anthropologist Geertz (1973), writing about the interpretation of cultures, argues that culture does not drive human behaviour. Rather, culture is a web of symbols that can be observed and thickly described to help us better understand what that behaviour means in a particular context. He points out that culture denotes:

[*A*]n historically transmitted pattern of meanings embodied in symbols, a system of inherited conceptions expressed in symbolic forms by means of which men [*sic*] communicate, perpetuate, and develop their knowledge about and attitudes towards life.’ (p. 89)

African philosophers view culture as an open-ended resource of social meanings on which members of a community draw to mediate the contingencies of their everyday life (Coetzee 2002). Culture denotes the resources of a community’s material and moral worlds, with ‘community’ defined as an ‘ongoing association of men and women who have a special commitment to one another and a developed (distinct) sense of their common life’ (Coetzee 2002:274). Culture has a normative role in a community. It consists of interpersonal standards for deciding how to go about daily activities based on the value placed on what it means to be a person, a human being, including what it means to be differently abled.

### Culture and disability

Writing about culture and disability, Ohajunwa and Sefotho (2024) point out that it is difficult to find a definition of disability that would be applicable to all cultures because conceptions of disability are linked to conceptions of ‘person’ and ‘personhood’ in a particular culture. In small-scale societies (i.e. local communities), close social interactions between individual members are the norm, with each individual having extended, multistrand relationships with other members of the surrounding cultural society. They observe that there has been epistemic exclusion of cultural positive experiences of disability both from the individual and social contexts. Beyond definitions, models of disability better explain the relationships between cultures and disability (Retief & Letšosa 2018). The global contemporary understanding of disability embraces epistemologies of disability from the global south, specifically focusing on indigenous disability studies (ed. Ward 2024). Contemporary debates focus on disability inclusion, the increasing awareness, interest in including disability and disability-oriented employment (Mullin et al. 2024). Diversity, equity and inclusion (DEI), corporate social responsibility (CSR) and the UN Sustainable Development Goals (SDGs) have become global guiding frameworks (Mullin et al. 2024). But these may not necessarily mirror Afrocentric ontoepistemologies of disability (Sefotho 2021). The social identity of persons with disabilities is based on inclusion in the family, clan and community life. In contrast, interpersonal relationships in large-scale societies (i.e. populations within and across countries) are impersonal. People with disabilities become a marked group; they are given social identity (as opposed to having one as members of a cultural group) by the State; they are viewed as citizens who have the same rights as others and should, as such, be included in all dimensions of mainstream society.

### Situating Ubuntu in development

An Ubuntu approach to disability and inclusive development foregrounds the benefits of mobilising people’s innate potentialities with due consideration of their cultural and communitarian orientations. Ramose (2002), writing about the philosophy of Ubuntu and Ubuntu as a philosophy, argues that Ubuntu is the fundamental ontological and epistemological category in the African thought of the Bantu-speaking people. The be-ing of an African in the universe is inseparably anchored upon Ubuntu. Ramose points out that Ubuntu is two words in one: the prefix ‘ubu-’ evokes the idea of a way of be-ing that is always orientated towards enfoldment of ‘-ntu’, towards humanity as kinship. Combined, the word Ubuntu represents two aspects of be-ing as oneness and an indivisible wholeness (Ramose 2002:230). It means ‘I am a person through other people. My humanity is tied to yours’. As a way of be-ing in the world, Ubuntu manifests humanness, it seeks to establish humane (polite, respectful, dignifying, consensual, compassionate) relations with others. Another way it manifests is as the ‘dance of be-ing’ (Ramose 2002:234). In this sense, Ubuntu is an invitation to participate actively in and through the music of being (Ramose 2002:235). Music, dance and storytelling are the means through which Africans search for harmony in all spheres of life.

Archbishop Tutu (1999), writing about commitment to a communitarian conception of the socially and culturally embedded personhood of all citizens in post-Apartheid South Africa, explained Ubuntu this way:

‘[*T*]he essence of being human is part of the gift Africa will give the world. It embraces hospitality, caring for others, being willing to go the extra mile for the sake of others. We believe a person is a person through another person, that my humanity is caught up, bound up and inextricable in yours. When I dehumanize you, I inexorably dehumanize myself. The solitary human is a contradiction in terms and, therefore you seek to work for the common good because your humanity comes into its own community, in belonging.’ (p. 34)

We argue that the importance of person-centred community based inclusive development (CBID) practice in the context of poverty and structural underdevelopment lies in working for the common good through consensual, participatory forms of empowered action-learning that draws on shared cultural values of Ubuntu.

### Human scale development

Human scale development (HSD) is a conceptual framework for a people-centred development practice that targets individual, group and community barriers to inclusive development by mobilising people’s various innate potentialities (Max-Neef 1991). Led by Chilean economist Manfred Max-Neef, an interdisciplinary team of Latin American researchers, formulated the HSD approach to improve quality of life, especially for poor people by focusing on the opportunities they have to satisfy their fundamental human needs. The HSD approach places emphasis on empowerment-orientated development processes at a human scale or local community level, rather than large national or international developments (Max-Neef 1991). It considers the nine fundamental human needs as universal, that is, they are the same in all cultures and historical periods, and satisfiers of these human needs may differ. See definitions of these human needs in Table 1.

**TABLE 1:** Definitions of fundamental human needs.

Need	Definition
Subsistence	The need for food, shelter, water – the things commonly referred to as ‘basic needs’, without which a person can die
Protection	The need to feel safe, secure, not scared that you are in danger
Affection	The sense that you are appreciated, accepted, loved; the need to have close friends or people whom you love and who love you
Understanding	The need to understand what is going on around you as well as the need to be understood by others
Participation	The need to be part of what’s happening, to belong to something, to take part in decisions that affect you; the need not to be isolated or ignored
Idleness	The need to rest, reflect, relax, take time out, play, do nothing
Creation	The need to be creative, to make things, invent things, use your own ideas and imagination
Identity	The need to feel that you yourself are important, that you are worth something, that you have something to offer
Freedom	The need to be free and not restricted; free to make your own choices and not have everything said and decided for you

*Source:* Lorenzo, T., 2005, ‘We don’t see ourselves as different: A web of possibilities for disabled women: How Black disabled women in poor communities equalise opportunities for human development and social change’, PhD thesis, University of Cape Town, Faculty of Health Sciences, Department of Public Health and Family Medicine, viewed April 2025, from https://hdl.handle.net/11427/9982; Max-Neef, M.A., 1991, *Human scale development*, The Apex Press, London

Besides subsistence, these fundamental human needs are not seen as hierarchical, but rather each need is regarded as equally important to human development. The HSD approach addresses poverty by meeting deeper human needs rather than just material aspects, economic goods or services. It seeks to develop self-reliance by overcoming human poverty in all its forms. In short, HSD targets human *poverties* in the belief that real development at an individual group or community level will only happen if all nine fundamental human needs are met.

For development to occur, each need is considered to have a twofold existential (lived experience) character that is held in constant tension. The resolution of this tension through a process of empowerment enables the gradual, progressive interruption and eventual elimination of the vicious cycle of poverty and underdevelopment. Firstly, each need has a *deprivation* dimension. Deprivation involves a psychophysiological response to an unmet need that is usually experienced as a feeling or sensation, an awareness that something appears to be missing. Secondly, each need has a *potentiality,* which indicates the degree to which the human need engages, motivates and mobilises people. Every potentiality can become an innate, existential resource through which development can unfold. The existential dimensions by which needs may be met (Lorenzo & Duncan 2020):

- **Being**: Through the way we feel or regard ourselves and the attributes and attitudes that are used to define one, whether individual or collective.
- **Having**: What we have – indicates institutions, customs, norms, mechanisms, non-material tools and laws (not physical facilities or goods and services).
- **Doing**: Through what we do – defines actions, whether individual or collective.
- **Interacting**: Where and with whom we interact – indicates collective locations or environments as settings in time and space.

Human scale development mobilises the human capacity of people to solve their own problems. It addresses scarcity of resources by helping people identify the creative and synergistic social potential that exists but lies dormant within themselves and their various environments. Limited resources can be stretched further when human needs are seen as life forces that mobilise people to act to meet their needs. A dynamic HSD matrix is created with human needs on the y-axis and existential dimensions on the x-axis (see Table 2). The HSD matrix serves as a template for identifying need satisfiers (Max-Neef 1991), that is:

the diverse range of means available to meet each fundamental human need. Satisfiers are culturally determined and vary according to time, place and circumstances. They can be seen in societal processes or practical ways or means that people adopt or choose to use to structure their lives. Singular satisfiers meet one need while synergistic satisfiers meet more than one need at a time. A satisfier is positive if it leads to beneficial and constructive outcomes. A satisfier is seen as negative in nature if its outcome is unhelpful or causes pathology, for example, violence. Societal and individual pathologies occur when needs are not met or when they are met in a negative way for a long period. (n.p.)

**TABLE 2:** An human scale development matrix of human needs and existential dimensions, with examples of satisfiers.

Fundamental human needs	Possible single or synergistic satisfiers			
	Having	Being	Doing	Interacting
Subsistence	Disability grants	Able to live healthily	Generate more income to create self-employment	Accessible facility to run own business
Identity	Access to cultural and traditional activities	Sense of belonging	Encourages inclusion and personal growth	Participate in cultural events with peers
Protection	Smartphones to create WhatsApp group	Being vigilant	Caring and prioritise safety	Attend community policing forums
Affection	Friendship circles through community notice boards	Belong to youth groups	Going out with friends, participating in sports	Attend community events and festivals
Understanding	Having sign language translator at community meetings	Treating others with respect	Teaching basic sign language to community people	Make information and communication accessible
Creation	Access to inclusive dancing	Being creative	Join dancing clubs that accommodate their needs	Attend shows at local theatres
Idleness	Accessible to open spaces like parks or beaches	Be by oneself	Read a book	Have time to self
Participation	Committees of different organisations	Be a committee member	Singing in a choir	Organise events or social groups
Freedom	Accessible transport available	Be confident to voice ideas, make decisions	Choose what to do	Participate in community events or programmes

*Source*: Mahlangu, J.M. & Lorenzo, T., in press, ‘Adopting three mindsets to community-based inclusive youth development’, in S. Grech & J.G. Weber (eds.), *Community-based inclusive development*, Chapter 5, Springer Nature

## Research methods and design

### Participant selection

The women who were involved in the PAR study lived in wooden shacks. A snowballing technique was used for selection. By the end of the study, 180 women had participated or were exposed to the project as part of CBID programmes. Any woman who met the following criteria was invited to attend the workshops:

- **Age:** between 18 years and 50 years.
- **Impairment status:** There were no exclusions related to any impairment category as a process of snowballing occurred. Participants had been referred by a primary health care organisation, South African Christian Leadership Assembly (SACLA) Primary Health Care Project, a non-governmental organisation (NGO) providing health and rehabilitation services in Khayelithsa and Browns Farm and KTC or Disabled People South Africa, by different routes, including self-referral. In this way, their status as ‘disabled’ was based on self-disclosure, that is, they identified with an experience of disability.
- **Economic status:** No one was excluded based on income, access to social security or employment. The women were low income, dependent on disability grants (DGs) and unemployed or self-employed doing informal trading.
- **Resident:** in the informal settlements served by two non-government organisations, one provided primary health care services, including the training of community health workers and mothers of children with disabilities as community rehabilitation workers; the other NGO was a national disabled person organisation.

### Data generation

Reflexivity as a research method is an intentional intellectual activity in which individuals explore or examine a situation, an issue, or object on the basis of their past experiences, to develop new understandings that will ultimately influence their actions or in which they critically analyse the field of action (Trembley et al. 2014:539). In re-examining references to Ubuntu in the synthesis chapter of Lorenzo’s (2005) PhD, we further theorised the relevance of an Ubuntu approach to disability inclusion that occurred through action spaces at local and community level. While CBID is applicable to both small-scale and large-scale societies, a ‘thick description’ is a criterion of ensuring rigour in qualitative research by describing the contextual factors of the study in depth, namely culture and disability is possible in small-scale contexts such as the community of women with disabilities in our study.

### Ethical considerations

Ethics approval was obtained from the Research Ethics Committee of the Faculty of Medicine, University of Cape Town on 14 May 1999 (REC Ref: #100/99). Pseudonyms were used when quoting data from participants to protect their identity.

## Results

Findings are presented in two parts. Firstly, the contribution of *Ubuntu* as an African philosophy in helping women resolve the tensions between the deprivations and potentialities of meeting their fundamental human needs. Secondly, the use of HSD as a conceptual framework for fostering community-based inclusive development.

### Part 1: Proposing an Ubuntu approach to disability inclusion as a framework encompassing interdependency and spirituality

**FIGURE 2:**
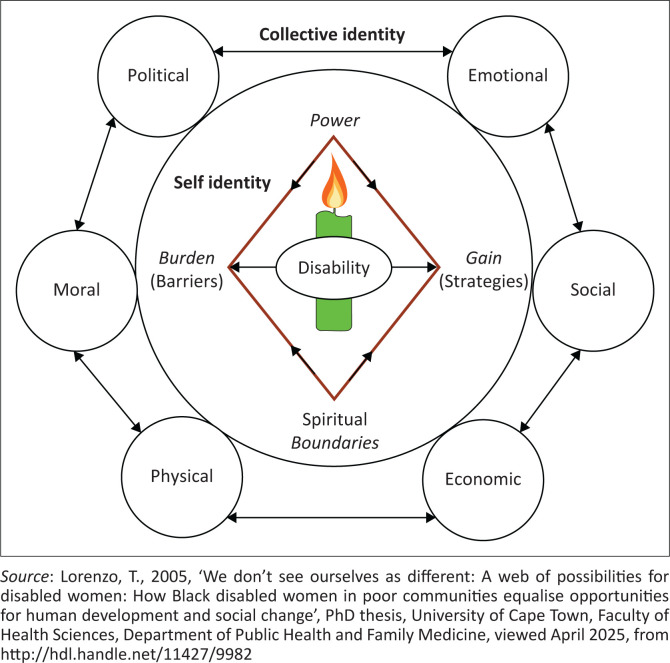
An Ubuntu approach to disability.

The findings suggested that the social model of disability has limitations in resource-constrained communities, where support to advance policy implementation is needed. The interdependency and spirituality reflected the women’s creative potential that emerged vibrantly during the NAR workshops. These propositions were evident in the women’s engagement with songs, dance and storytelling using indigenous symbols and metaphors to explain the existential dimensions of their human needs, that is, their shared cultural values and indigenous ways of being, having, doing and interacting. For example, women made clay sculptures of two cows grazing to illustrate the concept of ‘sharing with the other’. Their spirituality infused all the workshops through singing and rituals of prayers. Figure 2 depicts how Ubuntu, symbolised by a burning candle, provided spiritual impetus (light) to the relationships that formed during the facilitated action-learning processes of NAR workshops.

The body of the candle represents disability and the wick the potential of women to overcome the burden, if a catalyst, such as access to information, transport, support and dispelling myths, acts as a match to light the candle. As the light shines, it diminishes the darkness and extends the boundaries. Simultaneously, as the candle burns, the wax diminishes, in the same way that the burden of disability diminishes as the women overcome isolation, challenge and change stereotypes and create a network of action spaces through social reflexivity and collective action (Lorenzo 2005). The capacity of women, children, families and neighbours to understand disability issues is nurtured. As power increases, so do the women extend their boundaries for human development and vice versa. Human relationships and interactions are paramount during the action learning process– the essence of being a person through other people is illustrated in the different critical dimensions that comprise the collective identity (Lorenzo 2005):

‘I see myself as a light for other disabled people and I’m not afraid. I know I’m able to talk. I’m usually shy, but since I’ve been here I am free. I used to worry a lot at home, but since I’ve been here I’m much better. When I see my neighbours quarrelling, I say to them ‘Call your family and sort this out’. Later they say to me ‘Really we called them and we solved it’. So that is why I’m saying I’m a lamp. I see that even with the women, there is change. I’m able to see how they were before and how they are now. Ever since they have been meeting in the groups, the load was taken away and everything has been lighter.’ (Bulelwa, black person, physically disabled woman)

Figure 2 depicts the spaces and connecting lines between self-identity and collective identity that are dynamic, indicating the power of NAR workshops for promoting social reflexivity. Self-identity forms the inner circle because in African life, a person is the most important element of a society and forms the cornerstone of society as an inherent spiritual being. As such, a person is dependent on the goodwill and acceptance of others, which creates an inclusive society (Lorenzo 2005). Collective identity comprises six circles connected by two-way arrows to represent the interplay between the dimensions of human development, which include social, economic, political, as well as spiritual, emotional and moral development. The circles are all connected to the inner circle to indicate that no person can survive as an individual, whether disabled or not. *Disability is a burden or penalty* when it is experienced as a loss of power or imposed boundaries within society. *Disability as a gain or possibility* occurs when the myths of disability are dispelled and the boundaries of potential are extended (Lorenzo 2005). Talking about human poverty experienced at an individual level helped the collective to create a network of action spaces for their inclusion in opportunities for human development:

‘After the first workshop where I told my story, I felt much stronger. I realized these workshops could really help other women in the same situation as me. Our rehabilitation did not help us return and settle back with our families or communities. Here I made a clay sculpture of a plate and two women to share how I changed from gaining knowledge of disability rights and advocacy skills. The workshops helped us to find knowledge and information for each other. We felt happier. We recognized the gains we’ve made in changing our living conditions. These skills have led to a better life together. I made myself using clay. I want to show you that before I became disabled, my body was thin. I was small before and you can now see how big I am. So I want to share the good news so that others can be big like me. I must be the light, even in the community and preach about disability and how they can treat disabled people. I talk about disability. I became more confident and gained skills in being able to change things. I was used to speaking in church and sharing my testimony. Now I speak about disability. I also loved to sing and dance.’ (Bulelwa, black person, physically disabled woman)

Figure 2 also indicates that the vicious cycle of poverty and disability can be interrupted through the mobilisation of a collective, culturally embedded, people-driven development process at a local community level. The adverse cycles of deprivation in individual lives can be shifted by benevolent cycles of humanity and generosity (Ubuntu) in which the collective is stronger than the individual, and the individual strengthens the collective. From a sense of self-reliance and inner strength, the women were able to rise above their worries and anxieties and dream of a better life together. There was an intention to succeed in their endeavours. The women realised how they could use their time and energy more wisely and purposefully. They challenged each other to change their behaviours of self-pity and undermining their abilities, so as to increase their productivity. The changes echoed the characteristics of spiritual warriors (Roger 1998), which resembled the Ubuntu spirit of resilience (Tutu 2004). The sense of interdependency drew together the pain, hope and potential of women in the study, as women gained confidence to participate in activities to foster human development as they found support from each other. The spirit of compassion in advocacy reflected the women’s empathy for each other, as well as their neighbours:

‘So I was not the only one who wanted to shine a light to change attitudes to disability ourselves, amongst our families and our neighbours. The workshops also helped us see how we could heal each other. The workshops gave us courage to be visible in our families and community again. I told a story from the bible: I identify myself with the person who was next to the dam and people were coming and going not helping him. So Jesus asked him, “do you want to be well?” “I want to, but I don’t have a person who will help me and put me in this dam.” Jesus said “take your mat and go and by those words you are healed.” So now I’m well, but it’s sad when you see others having problems.’ (Bulelwa, black person, physically disabled woman)

The textual data of the NAR workshops were inductively coded and plotted against the nine fundamental human needs of HSD, presented in part 2.

### Part 2: Human scale development as a conceptual frame for people-driven, community-based inclusive development

Two themes emerged from using the HSD as a conceptual framework for meta-analysis of data: *Disability as a burden or penalty* (a deprivation) and *Disability as gain* or possibility (a potentiality). See Figure 1 that reflects the cyclical nature of the NAR workshops.

**FIGURE 3:**
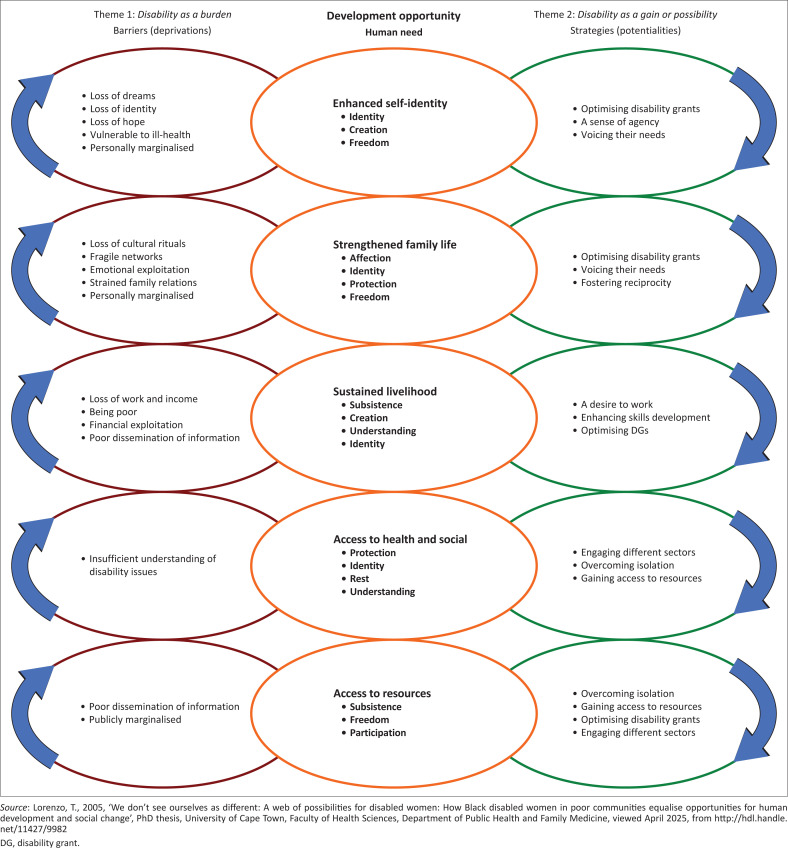
The push-pull tension between deprivations and potentialities of the human needs of women that emerged in the two themes: *Disability as a burden* and *Disability as a gain*.

Figure 3 illustrates the tension between the deprivation of unmet human needs and the potentiality of being able to meet one’s human needs through individual and collective actions motivated by the prevailing values of Ubuntu. As the collective action learning cycles progressed each woman, given her unique personal circumstances, gradually and at her own pace, began resolving the tensions in her unmet human needs, through individual or collective actions, culminating in progressive person-centred development. The data illustrated how a PAR process contributed to human-scale, people-driven, local community development, evidenced in enhanced self-identity, strengthened family life, sustained livelihoods, increased access to health and social services and access to information.

### Enhanced self-identity

During Phase 1, the women reported deprivations associated with impairment and ill health that resulted in a sense of powerlessness and hopelessness at an individual level. Their low confidence and self-esteem revealed human poverties of identity and understanding heard in the frequent statements of ‘I don’t know’. As the project progressed, gradual shifts in self-identity occurred that became a positive synergistic satisfier in situations where women were able to generate change through their sense of agency, well-being and wholeness:

‘I experienced deep changes about how I feel. I used to cry a lot, but since I met other women, I got new ideas. When Bulelwa introduced me to the group, I was happy and I became one of them. I understand more about this impairment now. When I became disabled, I always undermined myself when I was with my friends. I always sat in one place. But when I met with the other women I became stronger and that thing of always feeling sorry for myself is gone. My in-laws did not love me but today my house is always full. I’m strong. Today I’m not crying. Now no one can believe that I used crutches before. No one can say I’m disabled now. I identify myself with the sun because before it was dark and I didn’t want to accept my disability. I couldn’t even sit in the sun. Now I can do things with my hands and I got a certificate. After that I could do things for myself. Now I’m like a mother in the house even when the children are not there. I never used to be like that. I used to wait for them to come back from school. Now I feel that I can do anything.’ (Nomthandazo, black person, physically disabled woman)

### Strengthened family life

The women faced numerous deprivations in the context of family relationships, pressed by the scarcity of resources, both emotionally and financially. Some women were living with extended family members and were subject to abuse and exploitation. Others had family members who remained in the rural areas of the Eastern Cape, leading to a loss of family support and subsequent human poverty of affection and protection. Most women took on responsibilities of mothering as providers and nurturers, in some cases beyond the extended family to include others in the neighbourhood. Unemployment of family members put a strain on the women providing for the survival needs in the family using their disability grant (DG). Telling stories about their family-related deprivations gradually unlocked relational potentialities as family and public perceptions of the women being passive and dependent began to shift. Bulelwa reflected on the changes she herself and Glady’s experienced:

‘Another woman told how her children had found support from the workshops as well as they also felt the pressure to change the stigma of disability. Gladys said: ‘I’m receiving a lot of support from my family. My two children always show interest in what I’m doing. At school they used to be laughed at by other children because of my disability. But they told them that with their mother, they can’t see any disability, but they see a mother who is a role model and who can afford everything unlike [*non-disabled*] mothers who can’t afford the basics. You see [*non-disabled*] mothers who can’t even afford buying their children shoes. As mothers we learnt to teach our children respect and good social skills’. Gladys also challenged us: ‘Your child has a right to ask for things from you, but you need to teach them to talk nicely when they are asking for something. They mustn’t go and ask for something from other people because they think you can’t do things since you are disabled.’ (Bulelwa, black person, physically disabled woman)

### Sustained livelihoods

The women’s stories indicated that government efforts to provide education (primary, secondary or tertiary) to children and women, young and old, had not yet materialised at a grassroots level. They reported low levels of schooling and high rates of illiteracy due to missed opportunities for formal education because of their socio-economic circumstances in the historical context of racially segregated schooling. Some women generated income from vegetable gardening and selling in the informal sector that hardly enabled a subsistence existence because the small profit that was generated had to be used for school fees and buying children’s clothes. Gradual shifts in ideas for and actions towards livelihood generation emerged. Skills development for self-employment and finding a place to work were identified as synergistic satisfiers of human needs to equalise opportunities for subsistence. The human need for identity was met in resuming the provider role and the human needs of understanding and creation were met through learning new income-generating skills:

‘As a facilitator, the other women saw someone who is disabled and who at the same time was capable of sharing with them and of knowing what we should do together. We have gained a lot from each other. I gained strength from them; I gained just from sitting down with them, even if we were just talking. We found a woman who was in a different position when she first came in, in a different state. But today we are talking to someone else who is so grown. Often the woman herself doesn’t even realise it, but we saw how she came in, and the difference today. Women are also taking their businesses very seriously. Those who are starting small businesses have gained. They came in not knowing what they were going to do with their hands. Many had been sitting at home not knowing what to do. But today they are proud of what they are doing, having skills of many kinds. Some of the women are now working in the shops.’ (Majorie, black person, physically disabled woman)

### Community rehabilitation workers as brokers to access health and social services and information

Interconnections between the barriers to health and social services were evident in the findings. Most women interacted with health and social services as a consequence of their impairment. In public health services, because of insufficient understanding of disability issues and poor dissemination of information by practitioners, the women experienced difficulty in accessing DGs. Ironically, inadequate financial resources meant a sense of deep frustration was experienced when trying to access health and social services. The stories indicated that social security in the form of DGs and neighbourhood structures were identified as synergistic satisfiers of the human needs. The paradox of DGs is that they have the potential to enable women to assume a provider role and foster reciprocal support on the one hand (positive, synergistic satisfier). On the other hand, receiving a DG opened women to financial exploitation by family members (a negative satisfier). Participation of community rehabilitation workers (CRWs) in the PAR process paved the way for the women to appreciate the role of these mid-level health workers in facilitating increased access to intersectoral services and community-based rehabilitation. The support of CRWs provided the best chance of fostering skills and enhancing the abilities of the women to engage in occupations again. Based on effective referrals to support networks, some of the women were able to resume familiar roles and activities through community-based rehabilitation interventions. As a buffer between the women and service providers, the CRWs offered hope and direction to the women by meeting their initial need for support and information and by facilitating access to activities and resources. The CRWs, as mothers of children with disabilities, had walked a similar emotional path as the women. They were able to empathise and encourage women to take action to manage their resources to change their circumstances.

It became evident from the stories that poor dissemination and uptake of disability and development-related information could partially be attributed to high rates of educational illiteracy among the women, inadequate knowledge about disability among public sector service providers, inappropriate messaging by the media and uninformed cultural perceptions about disability in the public. The workshops created multilayered opportunities for gaining new, factual and accurate information and learning practical strategies for gaining knowledge and acting on them. As the women gained understanding, they experienced greater freedom to engage with information for more effective participation in development opportunities.

## Discussion

The findings support an Ubuntu approach to community-based inclusive development for women with disabilities that emerged using HSD (Max-Neef 1991) as a conceptual framework for the doctoral study. Two critical dimensions of this approach will be discussed: firstly, the creative dynamic between self-identity and collective identity as an impetus for social change and secondly, a spirituality of disability.

### The connection between self-identity and collective identities: interdependency as an impetus for social change

Figure 2 and Figure 3 allude to the complexity and interplay between self-identity and collective identity in the context of community-based inclusive developments. It is proposed that if the collective identity is ignored in human development initiatives, then the development of self-identity is hindered and vice versa. Both disability and identity are inherently complex concepts (Johnstone, Limaye & Kayama 2017). Disability self-identity can be perceived from an individual as well as societal levels (Lejzerowicz 2017), thereby presenting the idea of a dialogical self (Hermans 2012) as the nexus for an Ubuntu approach to disability and development. The findings indicate that disability identities need to be deconstructed as they are generally associated with deprivations such as stigma, isolation and self-limiting beliefs. A collective NAR journey helped the women with disabilities feel a sense of belonging. They felt at home again in their families and communities, that it was fine for them to be different and to openly talk about their disability without fear of stigma.

Gill (1997:39) identified ‘Four types of integration underlying disability identity development, delineated with examples: (1) “coming to feel we belong” (integrating into society), (2) “coming home” (integrating with the disability community), (3) “coming together” (internally integrating our sameness and differentness) and (4) “coming out” (integrating how we feel with how we present ourselves)’. These four types of integration were made possible by tapping into prevailing Ubuntu values. From the meaning of Ubuntu: *umuntu ngumuntu ngabantu* (isiZulu), similar to *Botho: Motho ke motho ka batho* (Sotho), ‘I am because we are’, is central to the concept of being human. The ‘self’ is equated to being human and extended or enriched through other humans as a fundamental interconnectedness of all human beings. The *Ubuntu-Botho* notions of self-identity are embedded in collective conceptualisations of a person in which a person is a person because of others and defined as equally human to everyone (Ntshangase 1994). Self-identity transcends the individual space into collective spaces where an individual possesses multiple identities. As the findings revealed, a woman with disabilities could be a wife, mother, daughter-in-law, aunt, sister, community member with certain roles and a plethora of other roles that make one’s self-identity (Lorenzo 2005).

When the interdependency of Ubuntu and the spirituality of disability were harnessed in the design of social change processes, the creation of supportive, inclusive communities was facilitated. The power of creative triggers and learning to listen deeply to each other’s stories evoked images of deprivations while illuminating the potentialities for the courage to act. Reason (1994), writing about the developmental benefits of action learning, comments:

‘A person is a fundamental spiritual entity, a distinct presence in the world, who has the potential to be the cause of his or her own actions. To actualise this capacity and become fully a person is an achievement of education and self-development … [*the process*] involves learning to integrate individualizing characteristics with a deeper communion with the others and the world.’ (p. 41)

Utilising creative methods that generate data about both individual and collective experiences of sustained social change enabled women with disabilities to drive their own development as opportunities for acting, reflecting, learning and planning generate productive action spaces. Understanding and applying HSD is a competence that could be embedded in the research curricula of undergraduate programmes at higher education institutions.

### The influence of spirituality of disability

The candle in Figure 2 foregrounds a spirituality of disability as ‘a driving force behind the women’s resilience and imminent collective identity’ (Lorenzo 2005:160). Insights into the spirituality of disability arising from this study have been reported elsewhere and will not be detailed here (Lorenzo & Duncan 2020). Suffice it to point out that Figure 2 positions power and spirituality as two interconnected dimensions for developmental shifts in self-identity and collective identity. Hull (2003), writing on definitions of spirituality and the defiant power of the human spirit, asserts:

Human spirituality is that which transfigures and transcends the biology of the human. When we speak of transcending the biological, we refer to those potentials of the human being which enable him or her to make the biological organism instrumental to non-biological purposes. These potentials include abstract thought, imagination, empathy, the ability to represent biological experiences symbolically, and the capacity to integrate experience and knowledge around a significance or a meaning which goes beyond the pleasure and pain of the individual. (p. 22)

The last sentence of this definition bears relevance to the spirit of our article. The findings linked to the existential dimensions of HSD (being, having, doing, interacting) support the centrality of spirituality in helping women with disabilities cope in the context of multiple deprivations. Lorenzo (2005:165–167) adopted Makgoba’s (1999) criteria of an African Renaissance as the potentialities relate to ‘means of asserting and promoting humanity, readiness [*of women with disability*] to question themselves [*with*] a permanent openness of mind [*for*] assimilation and adaptation’. Enabling a spirituality of disability during development processes allows abstract thought, imagination and empathy to turn the pleasure and pain of the individual into hopeful action (Lorenzo & Duncan 2020).

### Limitations

To claim that an Ubuntu ethos existed throughout the NAR processes is not necessarily to claim that the compassion it encapsulates was prevalent in the micro-dynamics of interpersonal relationships during each workshop. A limitation may be that there was no follow-up with women who discontinued participating in the workshops. Reasons could be mixed, as some women may have been satisfied, or they may have felt it was a waste of time as we were not meeting their needs or expectations. To the best of our knowledge, NAR workshops as a data generation method have not been utilised by other researchers, so its veracity has not been tested.

## Conclusion

This article highlights how NAR workshops as a data gathering method in Lorenzo’s doctoral study utilised creative art as triggers for dialogue and action spaces that made it possible for fundamental human needs to be met. The Ubuntu values that emerged during the synthesis of findings promote a reflexive, person-centred and collective approach to human development at the micro-level. Group interactions during NAR workshops made social reflexivity possible, fanning the flame of Ubuntu that was already embedded among participants. We have examined and theorised references to Ubuntu values in the doctoral research, highlighting the indigenous cultural processes that were elicited by the NAR workshops. The process of the women’s personal agency to act both individually and collectively was ignited through shared stories that generated a culture of belonging by raising awareness and consciousness about both the barriers to and opportunities for inclusive development. Developing action spaces to equalise opportunities was a synergistic satisfier of their fundamental human needs that disrupted the vicious cycles of poverty and disability. Interdependency and a spirituality of disability created a compassionate and sustained cycle of humanity and generosity, both significant values of Ubuntu, as action spaces facilitated people-driven inclusive development at a local level. The collective is stronger than the individual; the individual strengthens the collective.
